# Cardiovascular Benefit of Sodium-Glucose Cotransporter-2 (SGLT-2) Inhibitors in Type 2 Diabetes: A Systematic Review

**DOI:** 10.7759/cureus.18485

**Published:** 2021-10-04

**Authors:** Petros Georgiou, Wangpan Shi, Tatsiana Serhiyenia, Aqsa Akram, Matthew C Proute, Roshini Pradeep, Mina E Kerolos, Safeera Khan

**Affiliations:** 1 Department of Research, California Institute of Behavioral Neurosciences & Psychology, Fairfield, USA

**Keywords:** sodium-glucose cotransporter 2 inhibitor, diabetes type 2, cardiovascular disease, cardiovascular benefit, cardiovascular outcome trials

## Abstract

Cardiovascular disease (CVD) is the leading cause of mortality worldwide yet, despite advances in treatment, CVD remains an underestimated and undermanaged condition, with an even greater risk in Type 2 Diabetes Mellitus (T2DM). Sodium-Glucose Cotransporter-2 Inhibitors (SGLT-2i) are a promising novel drug class reported to improve Cardiovascular (CV) and renal outcomes in T2DM. Recent large-scale trials have assessed their CV safety with unexpected findings of multiple systemic benefits that could potentially reverse CVD. In this systematic review, we examined ten Randomized Controlled Trials (RCTs) that looked at cardiovascular outcomes in Type 2 diabetics and SGLT-2i. The RCTs were appropriately screened, looking for clear primary or secondary outcomes on CV events, and compared with placebo or other antidiabetic drugs. The RCTs had an average sample population studied of 5,549 participants with a mean follow-up time of 2.66 years. Three of the studies focused on CV parameters and risk factors. The remaining had defined CV composite events, and all consistently observed at least one CV benefit when using SGLT-2i. Our review of SGLT-2i in Type 2 diabetics showed the greatest benefit in reducing Heart Failure (HF) exacerbation and modest lowering of CV complications in high CV risk participants. Overall, there is still uncertainty about the exact mechanisms of SGLT-2i in their CV benefit, and whether they would favor pre-diabetic populations and those at earlier stages of CVD.

## Introduction and background

Every 17 seconds, a new diabetes diagnosis is made in the United States (US) [1]. Worldwide, it is one of the most prevalent chronic conditions, forecasted to affect 700 million adults by 2045 [2]. Diabetes, notably Type 2 Diabetes Mellitus (T2DM), is a polygenic disease that has a range of clinical severity. Its pathogenesis is closely linked to environmental factors such as obesity, diet, and smoking - with serious implications for accelerating cardiovascular disease (CVD). T2DM and CVD are part of the ‘‘common soil hypothesis’’ in that they share many risk factors - both environmental and genetic [3]. It is known that the most common cause of morbidity in T2DM are CVD sequelae such as myocardial infarction (MI), heart failure (HF), and stroke with T2DM individuals at equal risk of MI and stroke when compared to an individual with a prior MI or stroke history [4]. Obesity is the leading risk factor for developing T2DM and is an independent contributor to CVD through other conditions including hypertension, obstructive sleep apnoea, and dyslipidemia [5]. It can be anticipated that the need to address CVD will increase as obesity is projected to reach 50% of the adult population in the US by 2030 [6]. Our limitation of treatment is evident by CVD conditions like chronic HF, and accelerated coronary artery disease (CAD), being two to four times more likely to develop in individuals with diabetes [4].

The introduction of Sodium-Glucose Cotransporter-2 Inhibitors (SGLT-2i), licensed in 2013 for T2DM, has often been used as an adjunct alongside other diabetic medication in severe cases. However, several new suggested mechanisms of SGLT-2i may be the key for optimizing glycemic control, earlier on, and improving CVD outcomes. SGLT-2i works by blocking an important sodium-coupled glucose channel in the proximal tubule of the kidney. This promotes natriuresis with glucose loss that has multiple benefits - as seen in randomized controlled trials (RCTs) - including weight loss, lowering blood pressure, urate, and plasma volume. It has been observed that SGLT-2i has a unique role in reducing cardiovascular (CV) events, atherosclerosis, and exacerbation of heart failure (HF) [7]. This has been demonstrated further in a recent large-international trial of T2DM patients (the Asia Pacific, the Middle East, and North America) that SGLT-2i, even in patients without a CVD diagnosis, had a significantly lower risk of atherothrombotic events and death overall [8].

However, despite the promising effects of SGLT-2i and their cardiorenal protection, there is still disparity about their mechanisms for reducing CVD risk. We do not yet understand the systemic effects and the extent that SGLT-2i benefit diabetic patients - especially in the context of co-morbidities. Ongoing Cardiovascular Outcome Trials (CVOTs) have shown mixed data with degrees of significance [9]. It has prompted debate as to whether SGLT-2i cardiorenal protection is relevant in pre-diabetic populations, who would benefit most, and what are the reasons for their clinical benefit in the CVOTs [10].

## Review

In an aging population, diabetes will grow as one of the greatest global challenges. It already represents a major economic burden on healthcare systems with 9.4 times greater per capita spent on healthcare for diabetic complications [11]. Our systematic review aims to analyse the largest clinical trials to date to establish the level of known cardiovascular benefit from SGLT-2i. We will also evaluate the mechanisms proposed, and provide clarity on vulnerable subgroups that would benefit most from SGLT-2i intervention. The articles in this review were systematically screened and their methods and results are outlined.

Method

Our methods and results for systematic review are reported according to the Preferred Reporting Items for Systematic Reviews and Meta-Analysis (PRISMA) guidelines following our screening selection [12].

Search Strategy

We used electronic databases PubMed and Cochrane Library to look for articles using Medical Subject Headings (MeSH) and keywords to highlight the most relevant reviews and studies for analysis. The keywords included: ‘‘Diabetes Mellitus Type 2’’, ‘‘Sodium-Glucose Transporter 2 Inhibitors’’, ‘‘Cardiovascular Disease’’ and ‘‘Outcomes’’. We used the Boolean method to put together the keywords to an algorithm to use in PubMed. The articles were screened to highlight those most relevant to the search question and selected according to the inclusion/exclusion criteria.

Inclusion and Exclusion Criteria

The selection choice was purely from randomized control trials (RCTs) published from 2016-2021. All selected articles were peer-reviewed and published in the English language. Grey Literature was excluded. Our selection for eligibility followed the Population, Intervention, Comparison, Outcomes (PICO) model.

Data Extraction

The data retrieval and review were completed by two separate researchers independently. In the case of any disagreements, the researchers would discuss the data for its relevance and design to eligibility criteria to reach an accord. A third researcher was counseled for objectivity if a decision could not be met.

Critical Appraisal of Studies

We critically appraised our screened articles using the Cochrane risk of bias tool [13]. The bias risk assessment looked at seven causes of potential bias, and a summary was given for each clinical trial in this review in Table 1. 

**Table 1:** A summary of the RCTs bias using the Cochrane assessment tool RCT=Randomized Controlled Trial

COCHRANE APPRAISAL	Random Sequence Generation - Selection bias	Allocation of concealment - Selection bias	Blinding of both participants and evaluators - Performance bias	Blinding of assessment during outcome collection - Detection bias	Incomplete outcome data - Attrition bias	Selective reporting - Reporting bias	Other bias / Comments
Wanner et al. 2016 [14] EMPA-REG	LOW RISK	HIGH RISK	LOW RISK	LOW RISK	LOW RISK	LOW RISK	Small sample size to generalise to the broader population
Neal et al. 2017 [10] CANVAS	LOW RISK	LOW RISK	HIGH RISK	LOW RISK	LOW RISK	LOW RISK	Few clinical events, small proportion of participants to generalise
Wiviott et al. 2018 [7] DECLARE TIMI-58	LOW RISK	LOW RISK	LOW RISK	LOW RISK	LOW RISK	LOW RISK	Premature discontinuation of thousands of patients from each study group - statistical data possibly affected
Perkovic et al. 2019 [15] CREDENCE	LOW RISK	LOW RISK	LOW RISK	LOW RISK	HIGH RISK	LOW RISK	Trial stopped early - potential limited power
Phrommintikul et al. 2019 [16]	LOW RISK	LOW RISK	LOW RISK	LOW RISK	LOW RISK	LOW RISK	Small sample size
Bonora et al. 2019 [17]	LOW RISK	LOW RISK	LOW RISK	LOW RISK	UNCLEAR	LOW RISK	Small sample size
Petrie et al. 2020 [18] DAPA-HF	LOW RISK	LOW RISK	LOW RISK	LOW RISK	LOW RISK	LOW RISK	N/A
Fuchigami et al. 2020 [19]	LOW RISK	LOW RISK	LOW RISK	HIGH RISK	LOW RISK	LOW RISK	Small sample size, poor genetic diversity (all Japanese), short follow up
Packer et al. 2020 [20] EMPEROR-REDUCED	LOW RISK	LOW RISK	LOW RISK	LOW RISK	LOW RISK	LOW RISK	N/A
Cannon et al. 2020 [21] VERTIS-CV	LOW RISK	LOW RISK	LOW RISK	HIGH RISK	LOW RISK	LOW RISK	N/A

Results

**Figure 1:**
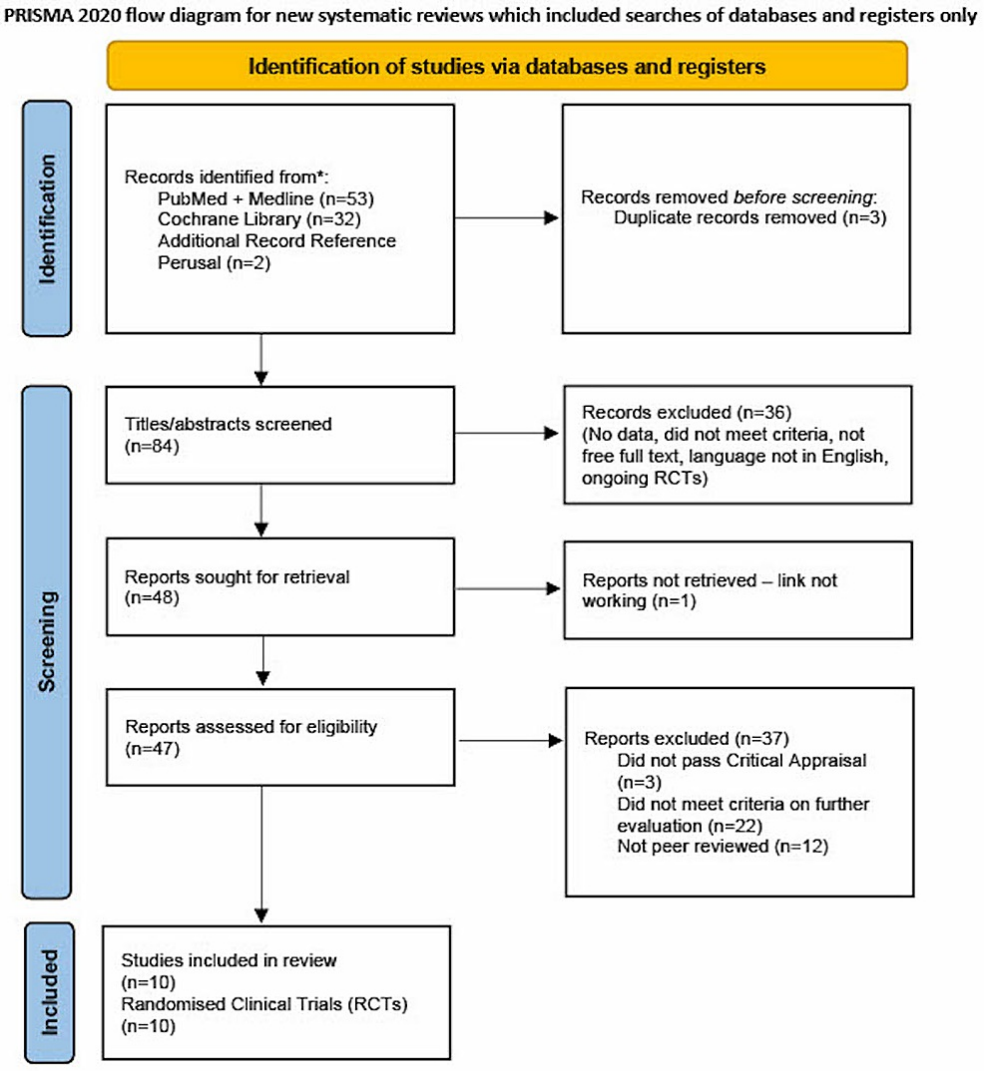
PRISMA 2020 flow diagram PRISMA=Preferred Reporting Items for Systematic Reviews and Meta-Analyses [12]

A total of 87 articles were generated from keywords, eligibility criteria, and databases. Of the 87 articles, 53 were from PubMed, 32 from Cochrane Library, and two articles were obtained from references to relevant journals. Duplicates were removed and 84 were screened from their titles and abstracts. A further 36 were discarded due to topic irrelevance. Of the remaining 48, one was removed for the link not working, three for not passing the critical appraisal, 22 for not meeting the criteria, and 12 for not being peer-reviewed. 10 articles met the criteria and were only RCTs. Our PRISMA flow diagram is shown below in Figure 1.

All the reviewed clinical trials differed in design, population, and primary endpoints. However, the study of SGLT-2i and Major Adverse Cardiovascular Events (MACE) was a common part of each RCT. This is shown in a summarized table below in Table 2.

**Table 2:** An outline summary of the clinical trials (n = number of patients in RCT) RCT=Randomized Controlled Trial, MACE=Major Adverse Cardiovascular Events, CVD=Cardiovascular Disease, RF=Risk Factors, MI= Myocardial Infarction, HF= Heart Failure, eGFR=Estimated Glomerular Filtration Rate, ESRD=End Stage Renal Disease, BMI=Body Mass Index, ACEi=Angiotensin Converting Enzyme Inhibitors, ARB=Angiotensin Receptor Blockers, T2DM=Type 2 Diabetes Mellitus, HbA1c=Glycosylated Hemoglobin Type A1C, HDL=High Density Lipoprotein, SBP=Systolic Blood Pressure, HFrEF=Heart Failure with Ejection Failure, NYHA=New York Heart Association, LVEF=Left ventricular ejection fraction, NT-proBNP=N-Terminal pro B-type Natriuretic Peptide, ICD=Implantable cardiac defibrillator.

COCHRANE APPRAISAL	Study Design	Inclusion criteria	Intervention	Primary Outcome	Secondary Outcome	Conclusions
Wanner et al. 2016 [14] EMPA-REG	Multicentre randomized double-blind placebo-controlled trial	Insufficient glycemic control and High risk of CV events N = 7020	Empagliflozin 10mg, Empagliflozin 25mg or Placebo	First occurrence of MACE (3-point) which included death from CV causes, non-fatal MI, or nonfatal stroke.	Expanded occurrence of MACE to include unstable angina as well as HF exacerbation, renal events and transient ischemic attack	Primary outcome - significant lower risk of MACE in the empagliflozin group than in the placebo group
Neal et al. 2017 [10] CANVAS	Randomized double-blind placebo-controlled	Male or female T2DM ≥ 30yrs with symptomatic CVD or 50yrs or older with two or more RF for CVD N = 9734	Canagliflozin 100mg, Canagliflozin 300mg or Placebo	Composite of death from CV causes, nonfatal MI or nonfatal stroke	Death from any cause, from CV cause, progression of albuminuria and composite of death from hospitalization for HF	Pt. with T2DM with Canagliflozin had lower risk of death from CV cases, nonfatal myocardial infarction, nonfatal stroke than placebo
Wiviott et al. 2018 [7] DECLARE TIMI-58	Randomized double-blind placebo-controlled	Male or female T2DM ≥ 40 yrs with T2DM and High risk for CV Events N = 17160	Dapagliflozin 10mg or Placebo	Composite endpoint of CV death, MI, Ischemic stroke or Hospitalization due to HF	Renal Composite endpoint - ≥40% decrease in eGFR to eGFR <60 ml/min/1.73m2 and/or ESRD and/or Renal or CV death	Pt. with T2DM with treatment with dapagliflozin did not result in higher or lower MACE rate than placebo but did result in lower rate of CV death or hospitalization for HF.
Perkovic et al. 2019 [15] CREDENCE	Randomized double-blind placebo-controlled	T2DM with HbA1c ≥6.5 and ≤12% with eGFR ≥30 and ≤90, Pt. need to be on maximum tolerated dose of ACEi or ARB 4 weeks prior to randomization, urine albumin to creatinine ratio >300mg/g and <5000mg/g N = 4401	Canagliflozin 100mg or Placebo once daily	Composite of doubling of serum creatinine, ESRD and renal or CV death	Composite Endpoint of CV death and Hospitalized HF	Pt. with T2DM and kidney disease, the risk of CV events was lower in the canagliflozin group than placebo
Phrommintikul et al. 2019 [16]	Prospective randomized double-blinded study	Adults ≥21 or non-childbearing potential female, inadequately controlled T2DM with at least half maximum dose of metformin. Stable documented coronary artery disease N = 49	Dapagliflozin 10mg or Vildagliptin 50-100mg	Individuals who have ST segment depression, average SBP, myocardial dysfunction, myocardial injury, oxidative stress, ventricular wall stretch and inflammation event.	N/A	Cardiovascular benefits observed only in SGLT-2i
Bonora et al. 2019 [17]	Randomized single blind placebo control	Male and Female aged 18-75yrs, T2DM on oral agents +/- insulin, with T2DM duration > 6 months with HbA1c 7-10% N = 33	Dapagliflozin 10mg or Placebo	Change from baseline in reverse cholesterol transport, measured as cholesterol efflux capacity of patient's plasma	Change from baseline in HDL cholesterol levels, Changes from baseline in the distribution in HDL subclasses, and adverse events	Observed no changes in cardiac function parameters estimated by impedance cardiography in T2DM.
Petrie et al. 2020 [18] DAPA-HF	Randomized quadruple blinded placebo-controlled	Male or Female aged ≥18yrs, diagnosis of symptomatic HFrEF (NYHA class II-IV) present for 2 months, LVEF ≤40%, elevated NT-proBNP eGFR ≥30mL/min/1.73m2 N = 4742	Dapagliflozin 5mg, 10mg or Placebo	Composite endpoint of CV death, hospitalization due to heart failure or due to HF	Recurrent hospitalizations due to HF and CV death, composite endpoint of ≥50% sustained decline in eGFR, ESRD or Renal Death, endpoint of all-cause mortality	Dapagliflozin when added to recommended therapy significantly reduced the risk of worsening HF or CV death independent of diabetes status
Fuchigami et al. 2020 [19]	Randomized parallel open blinded study	Male and female pt. who are ≥20yrs but ≤80yrs, T2DM, no antidiabetic medication or only using beguanide, HbA1c ≥7.1%, BMI ≥23kg/m2 N = 340	Dapagliflozin 5mg or Sitagliptin 50mg	Achieving HbA1c ≤ 7%, Body weight loss of 3% and avoidance of hypoglycaemia	Reduction in fasting blood glucose, body weight, BMI, lipid metabolism marker and other cardiovascular parameters	Compared to sitagliptin, dapagliflozin was more effective at improving cardiometabolic RF
Packer et al. 2020 [20] EMPEROR-REDUCED	Randomized double blinded placebo-controlled	Male or female ≥18yrs, pt. with chronic HF and elevated NT-proBNP, use of medical devices such as ICDs N = 3730	Empagliflozin 10mg or Placebo	Time to first event of CV death or hospitalization for HF	Total number of HF events, eGFR slope of change from baseline, all-cause mortality, all-cause hospitalization	Empagliflozin group had lower risk of CV or HF hospitalization than placebo group whether diabetic or not
Cannon et al. 2020 [21] VERTIS-CV	Randomized double-blind placebo-controlled	T2DM diagnosis, HbA1c 7-10.5%, on stable anti-hyperglycaemic agents, BMI ≥18, hx of atherosclerosis or cerebro/peripheral vascular disease N = 8238	Ertugliflozin 5mg, 15mg or Placebo	Time to first MACE, change from baseline in HbA1c% at week 18, change from Haemoglobin baseline at week 18	Time to occurrence of CV death or HF hospitalization, composite of renal death, renal dialysis/transplant, or doubling serum creatinine	Pt. with T2Dm and atherosclerotic CV disease, ertugliflozin was not significantly different for MACE when compared to placebo

Out of the 10 RCTs chosen, seven were double-blinded, one was quadruple blinded, with the remaining two RCTs open [19] and single-blinded [17]. The primary outcomes were a mixture of the timing of MACE, a composite endpoint of CV death, hospitalization due to HF, renal function, HbA1c reduction, and changes in cholesterol baseline. The majority of the large-scale trials also investigated for secondary outcomes that included a reduction in a range of renal, stroke, and hospitalization events. These secondary outcomes varied from mild to moderate beneficial changes that were statistically significant alongside the primary outcomes [20]. 

The populations being studied included adult males and females with set and defined CV risk factors or already established CVD [18]. The majority of RCTs were composed of T2DM participants with poor glycemic control that were screened with different parameters according to their renal function, HbA1c, medication status, BMI, CV risk or due to a combination of these. The SGLT-2i used in RCTs differed in dose and type and included Empagliflozin, Dapagliflozin, Canagliflozin and Ertugliflozin.

Three of the RCTs had significantly smaller population studies (less than 400), which is important to consider with their respective study conclusions. ​​​​​​In terms of primary outcomes, from Table 2, it is evident that the RCTs have yielded varying results when assessing cardioprotective effects of SGLT-2i. Bonora et al. 2019 [17], did not show any change in cardiac function, and the DECLARE-TIMI [7] trial showed cardiovascular benefit only in reduction of hospitalization from HF and CV death. The VERTIS-CV trial [21] also showed no significant change in MACE events or CV death in participants taking the SGLT-2i relative to placebo.

SGLT-2i across all RCTs did not demonstrate poorer primary CV outcomes when compared to the placebo. 

However, with the exception of the VERTIS-CV trial [21], the other six larger scale RCTs (population size greater than 3000) observed a primary outcome beneficial reduction in MACE events or CV hospitalization. The reduction was noted particularly for HF exacerbation, including RCTs that included diabetic and non-diabetic individuals [20]. The RCTs also had relatively few participants lost to follow-up from the administration and tolerability of the drug. The mean time for follow-up of the seven large-scale RCTs was 2.66 years. They show SGLT-2i are non-inferior to placebos. The most significant cardioprotective effects were seen in patients already at a high risk of cardiovascular events and severe HF - noted in EMPEROR-REDUCED by the percentage of ejection fraction and those who had a history in the last 12 months of HF exacerbation and level of N-terminal brain natriuretic peptide (BNP) [20].

Discussion

In order to bring new questions and conclusions in this systematic review, we will analyze the differences in RCT outcomes, the true cardiovascular benefit in T2DM, as well as the study limitations and potential mechanisms of SGLT-2i. 

Cardiovascular Benefits of SGLT-2i and Limitations

The RCTs in this review have shown cardiorenal protection in T2DM patients with SGLT-2i not currently seen in other anti-diabetic drugs. EMPA-REG 2016 trial, 7028 patients, all at high risk of CVD, reported a reduction in death due to CV causes in the SGLT-2i group (empagliflozin) vs placebo. This finding is reinforced by the CANVAS 2017 trial, with 9734 subjects reporting fewer rates of Major Adverse Cardiovascular Event (MACE) in the SGLT-2i group (canagliflozin) than placebo [10].

Across the large scale RCTs, the most significant cardiovascular benefit seen collectively in these RCTs has been a reduction in the effect on HF hospitalization - most notably in the CREDENCE [15] and EMPEROR-REDUCED [20] with CREDENCE specifically for HF hospitalization reporting a hazard ratio of 0.61; 95% CI, 0.47-0.8 P <0.001. The EMPEROR-REDUCED [20] has confirmed the DAPA-HF [18] findings where, in a cohort of 3730, lower heart failure hospitalization was considerable in the SGLT-2i group with a CV history (i.e., individuals with the previous MACE). It is evident that this benefit is amplified in T2DM and patients with chronic kidney disease (CKD).

**Figure 2:**
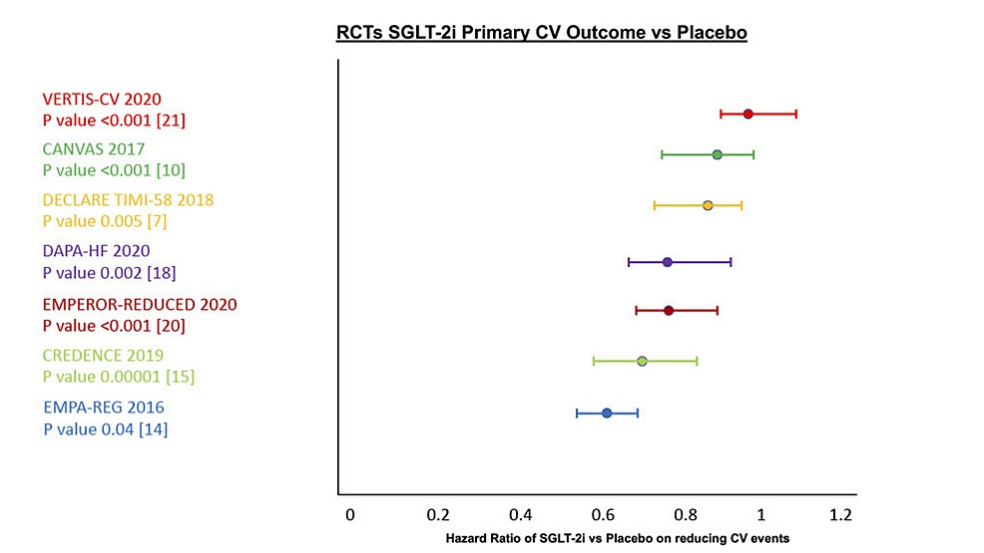
Cardiovascular Outcomes Hazard Ratio vs Placebo RCT=Randomized Clinical Trial, CV=Cardiovascular, SGLT-2i=Sodium-Glucose Cotransporter-2 Inhibitors RCTs - VERTIS-CV [21], EMPA-REG [14], CANVAS [10], DECLARE TIMI-58 [7], DAPA-HF [18], CREDENCE [15], EMPEROR-REDUCED [20]

However, with such differing RCTs in study design and criteria, we decided to review the large-scale RCTs in isolation from the smaller scale homogenous trials to establish the true cardiovascular benefit from SGLT-2i vs placebo outlined below in Table 3 and Figure 2.

**Table 3:** A summary of cardiovascular outcomes in SGLT-2i vs Placebo RCT=Randomized Controlled Trials, CI=Confidence Interval, MACE=Major Adverse Cardiovascular Events, CV=Cardiovascular, MI=Myocardial Infarction, HF=Heart Failure

RCT vs Placebo	Population studied	Intervention	Median follow up	Primary Outcome measured	Hazard Ratio for Primary Outcome	95% Confidence Interval (CI) and P value
EMPA-REG 2016 [14]	7020	Empagliflozin 10mg, 20mg vs Placebo	3.1 years	Composite of Cardiovascular death	0.61	95% CI 0.55-0.69 P value <0.001
CANVAS 2017 [10]	9734	Canagliflozin 100mg, 300mg vs Placebo	2.43 years	MACE	0.86	95% CI 0.75-0.97 P value <0.001
DECLARE TIMI-58 2018 [7]	17160	Dapagliflozin 10mg vs Placebo	4.2 years	Composite of CV death or HF hospitalization	0.83 (lower HF hospitalizations)	95% CI 0.73-0.95 P value = 0.005
CREDENCE 2019 [15]	4401	Canagliflozin 100mg vs Placebo	2.62 years	Composite of end-stage kidney disease or death from renal or CV causes	0.7	95% CI 0.59-0.82 P value = 0.00001
DAPA-HF 2020 [18]	4742	Dapagliflozin 5mg, 10mg vs Placebo	1.5 years	Composite of worsening HF or CV death	0.75	95% CI 0.63-0.9 P value 0.002
EMPEROR-REDUCED 2020 [20]	3730	Empagliflozin 10mg vs Placebo	1.3 years	Composite of CV death or HF hospitalization	0.75	95% CI 0.65 to 0.86 P value <0.001
VERTIS-CV 2020 [21]	8238	Ertugliflozin 5mg, 15mg vs Placebo	3.5 years	MACE	0.97	95% CI 0.85-1.11 P value <0.001

It is evident, from Figure 2, that the true cardiovascular benefit is overall promising with statistically significant reductions in primary CV outcomes in six of the seven large-scale RCTs. The most recent VERTIS-CV trial did not show a cardiovascular benefit in SGLT-2i, and the difference is likely multifactorial.

Firstly, there is a degree of unknown as to how SGLT-2i exert their systemic beneficial effects and whether this is merely due to chance. This is a crucial debate on SGLT-2i mechanisms that is masking the true long-term benefits of this drug class. Secondly, there is a dissimilarity among the CVOTs due to the population profile, the inclusion and exclusion criteria, and the specified endpoints that did not all statistically measure HF hospitalization like in the VERTIS-CV trial [21]. Thirdly, the difference in primary outcomes can be partly attributed to distortion, especially in recent studies where patient cohorts have potentially been receiving more rigorous CVD prevention therapies. The increasing use of medications such as anticoagulants, biguanides, and statins means that patients generally have been experiencing secondary prevention for a longer period - thereby underestimating the true benefit of SGLT-2i in isolation [22,23].

We decided to not include the three smaller trials for true CV benefit evaluation because their data was founded on sample sizes of less than 50 patients, with short follow-up times, that may have underestimated the long-term effects of SGLT-2i on cardiovascular function and remodeling [16,17]. The third smaller trial showed promising findings of SGLT-2i vs sitagliptin but the data stemmed from patients only in Japan [19]. This is important to consider, given the large genetics at play in CVD, when conducting future research to recruit a large heterogeneous sample.

The CVOTs primary and secondary outcome data indicate that the renal system plays a significant role in SGLT-2i beneficial outcomes. This can underestimate the benefit of SGLT-2i when only small proportions of patients with established kidney disease are included or when patients with severe kidney disease are excluded [7,10]. At the same time, this is where new research needs to expand on the impact SGLT-2i have in patients without established CV or renal disease, as many patients in RCTs benefitted despite not having a history of CVD or CKD. To optimize our understanding of SGLT-2i on CVD, the relative proportion and severity of individuals with established CVD should be more closely outlined in future RCTs, and a thorough CV history from participants is pivotal to minimize the potential for any confounders [20].

Despite the positive findings from six of the CVOTs for reducing CV events, there is insufficient data to extrapolate these findings to the general population [10,14]. The mean follow-up time across the seven CVOTs was 2.66 years. In order to ascertain the long-term benefits of SGLT-2i, subsequent research should either follow up T2DM participants over a longer period of time, or would need to ensure a large enough and relevant population (CV and T2DM risk) pool to extrapolate to the general population. The long-term benefits of SGLT-2i should be aimed at comparing the rates of micro- and macrovascular complications in T2DM.

New research should focus on minorities more at risk of CVD e.g., African Americans and Hispanics. These cohorts with T2DM should be further evaluated to see the effects of SGLT-2i and would provide more data to help represent the true population. Other significant cardiovascular outcomes have included a reduction in cardiac risk of death in patients without a diagnosis of diabetes [20]. This drives a need for future research to explore and evaluate the true benefit of SGLT-2i in individuals at risk of CVD or individuals who are pre-diabetic. It was estimated that 88 million adults had pre-diabetes in the US alone in 2018 with an estimated 18.2 million adults having a degree of coronary heart disease - the most common type of CVD and closely linked with diabetes [24,25]. 

Our limitations in this review were being unable to comment on the long-term CV benefits of SGLT-2i as the majority of data from the CVOTs are not applicable to the general population, based on patient demographic and strict T2DM status, and more ongoing data from current trials are warranted before a wide conclusion can be drawn. We also did not fully evaluate the adverse side effects of SGLT-2i as the safety profile of SGLT-2i has been well documented, and there was minimal to add from the CVOTs. The majority of adverse side effects in the CVOTs commented on genital and urinary infections, being greater in the SGLT-2i group than placebo, and levels of diabetic ketoacidosis being slightly higher in patients on SGLT-2i. There was data conflict as to whether SGLT-2i increased the risk of amputations or bone fractures [7,10,15].

We would hope that in a longer-scale trial with a larger population that these safety concerns could be better assessed. In general, it has been regarded as a relatively safe drug to use, and close monitoring can be easily achieved. 

Overall, SGLT-2i have shown significant CV benefit, the greatest benefit of reducing hospitalizations, CV death, and HF exacerbations compared to MACE. This could mean earlier use of SGLT-2i in patients with recent T2DM diagnosis or HF as these are progressive diseases. The use in non-diabetics or pre-diabetics with risk factors for CVD or CKD should also be strongly considered, and should be a focal point for future trials. 

Mechanism of Action of SGLT-2i

There are many proposed SGLT-2i mechanisms though what is less known are the primary mechanisms SGLT-2i exert their CV benefits. Certain accompanying factors have been proposed for explaining how they exert protective effects for specific CVD like HF exacerbation [26].

It is agreed upon that SGLT-2i in T2DM works by inhibiting the sodium-glucose transporter in the proximal tubule of the kidneys, reducing the amount of sodium and glucose reabsorbed, and promoting natriuresis and glycosuria. However, hyperglycemia has not been shown to be a strong or a reliable risk factor for CVD and would not explain the CV benefit seen acutely in both diabetic and non-diabetic patients [27].

The natriuresis effect has been shown to increase sodium excretion by up to 60% and this is thought to reduce the afterload on the heart and improve ventricular cardiac function [28]. However, the blood pressure effect shown from the CVOTs is modest and is unlikely to be the main reason for lower CV risk [14,20]. Clinical trials have speculated on the diuretic effect of SGLT-2i, and it has been shown to decrease albuminuria, plasma volume, and this has been thought to be the main driver in reducing HF exacerbation [10]. A suggested explanation for SGLT-2i has been that a reduction in plasma volume, intraglomerular pressure, interstitial volume, and an increase in erythrocyte mass are key to SGLT-2i systemic effects - not seen in loop and thiazide diuretics [29]. 

The CVOTs have seen a consistent reduction in cardiovascular HF hospitalization, the most notable in the DAPA-HF and the EMPEROR-REDUCED trial that had a reduction in HF Hazard Ratio of 0.7; 95% CI 0.58-0.85 P<0.001 [20]. Poor cardiac function is thought to lead to dysfunctional energy metabolism such as mitochondrial oxidative phosphorylation, making the heart more dependent on glycolysis [30]. SGLT-2i have demonstrated an increase in ketogenesis from adipose tissue thereby providing alternative energy for the heart and restoring levels of adenosine triphosphate (ATP). This suggested mechanism has yet to be evaluated in humans and should be a point of reference for future research [31].

While other CV events are less clear in terms of inflammation, HF severity is correlated with increased inflammatory biomarkers. There is not a clinically proven SGLT-2i drug class as superior to one another however, empagliflozin, canagliflozin, and dapagliflozin have shown benefit in regulating the immune system by downregulating extracellular matrix processes and levels of fibrosis [32]. This likely helps reduce Reactive Oxygen Species (ROS) and improve contractile function. The SGLT-2i cardioprotective effects are also suggested to be chronic inflammatory modulation of many processes such as macrophage response and specifically the nucleotide-binding oligomerization domain, leucine-rich repeat, and pyrin domain-containing 3 (NLRP3) inflammasome that is likely to help prevent cardiac remodeling [33].

Looking at the EMPA-REG outcome trial which raises the possibility of myocardium effects, further analysis showed a decrease in the Left Ventricular (LV) mass index of participants on empagliflozin vs placebo. This raises new research opportunities to assess the extent of reversibility, and the impact this has on cardiac function in the long term [14,34,35]. Much of the renal cardiorenal benefits in this trial continued to improve after discontinuation, indicating that SGLT-2i may have greater potency and reversibility than previous thought.

The DAPA-HF data demonstrates this observation of SGLT-2i independent of diabetes and the potential increase in erythropoietin (EPO) that could be behind improving renal function [18]. Key areas of study need to look further into SGLT-2i on sympathetic nervous system reduction, the extent to which failing hearts use ketones as secondary fuel, and the inflammatory process that raises the CV risk profile.

**Figure 3:**
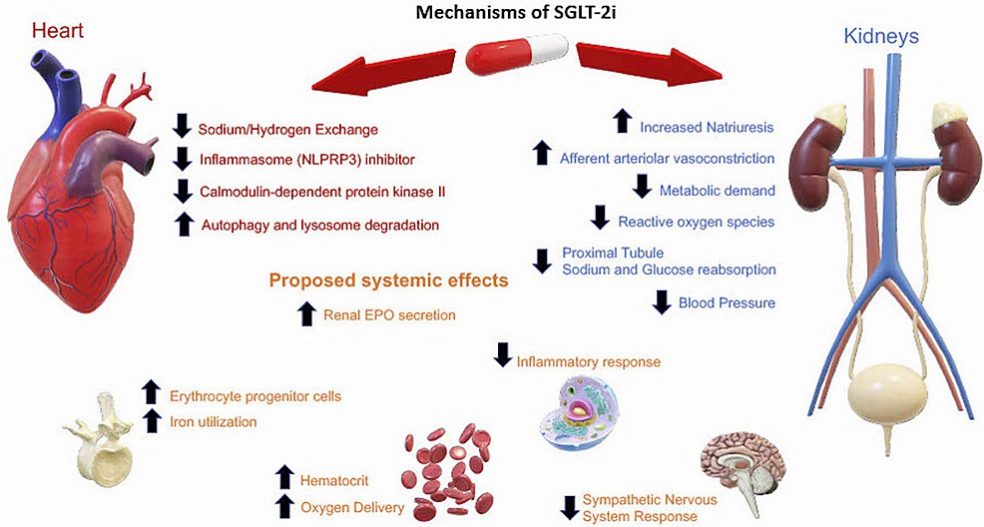
SGLT-2i Mechanisms of systematic and cardiorenal effects SGLT-2i=Sodium-Glucose Cotransporter-2 Inhibitors, NLPRP3=Nucleotide-binding Oligomerization Domain, Leucine-rich Repeat and Pyrin Domain-containing 3, EPO=Erythropoietin

Looking at the data from CVOTs, as well as experimental data, we can infer that the CV benefits are due to direct prevention of pathologic cardiac remodeling and indirectly by renal protection. SGLT-2i likely exert their indirect systemic effects by promoting afferent arteriolar constriction, reducing renal stress and intraglomerular pressure, and this lowers metabolic demand on the cardiorenal system and ultimately helps to maintain contractility. The proposed mechanisms known so far are summarised in Figure 3.

## Conclusions

SGLT-2i have proven to be a relatively well tolerated and safe drug - in the doses studied in the CVOTs - with the majority reducing the risk of HF exacerbation and hospitalization in T2DM. There was a moderate effect in lowering the risk of composite death from all CV causes, although there were mixed data likely due to limited homogenous sample size populations, and cohorts in recent trials already receiving extensive CV prevention therapy. A more interesting observation was the same cardiovascular benefit seen in patients with and without diabetes, raising the notion of SGLT-2i mechanisms being more independent regarding glycemic control.

Our systematic review brings in a wider scope of data focused on cardiovascular benefits. It shows that the favorable mechanisms of SGLT-2i are still unclear, and this should be a point for future research to study the extent that SGLT-2i prevent cardiac remodeling, the direct and indirect effects they have on the heart, and the relationship to whether the clinical benefits are largely from renal protection. The main limitation of our study is not being able to evaluate the true long-term adverse side effects of SGLT-2i, and the long-term benefit-risk in vulnerable individuals. This is vital to address in future interventional studies with longer follow-ups for participants. We should aim to examine a larger sub-group population to assess SGLT-2i in genetically higher risk CV groups, individuals with recent T2DM or HF diagnosis, and the effects in pre-diabetics. This will help us analyze more clearly the likely beneficial mechanisms of SGLT-2i and its application to the general population.

## References

[1] Fakorede FA (2021). Increasing awareness about peripheral artery disease can save limbs and lives. Am J Manag Care.

[2] Saeedi P, Petersohn I, Salpea P (2021). Global and regional diabetes prevalence estimates for 2019 and projections for 2030 and 2045: results from the International Diabetes Federation Diabetes Atlas, 9th edition. Diabetes Res Clin Pract.

[3] Bellastella G, Scappaticcio L, Esposito K, Giugliano D, Maiorino MI (2018). Metabolic syndrome and cancer: "The common soil hypothesis". Diabetes Res Clin Pract.

[4] De Rosa S, Arcidiacono B, Chiefari E, Brunetti A, Indolfi C, Foti DP (2018). Type 2 diabetes mellitus and cardiovascular disease: genetic and epigenetic links. Front Endocrinol (Lausanne).

[5] Cercato C, Fonseca FA (2019). Cardiovascular risk and obesity. Diabetol Metab Syndr.

[6] Ward ZJ, Bleich SN, Cradock AL (2019). Projected U.S. state-level prevalence of adult obesity and severe obesity. N Engl J Med.

[7] Wiviott SD, Raz I, Bonaca MP (2019). Dapagliflozin and cardiovascular outcomes in type 2 diabetes. N Engl J Med.

[8] Kosiborod M, Lam CS, Kohsaka S (2018). Cardiovascular events associated with SGLT-2 inhibitors versus other glucose-lowering drugs: the CVD-REAL 2 study. J Am Coll Cardiol.

[9] Lo KB, Gul F, Ram P, Kluger AY, Tecson KM, McCullough PA, Rangaswami J (2020). The effects of SGLT2 inhibitors on cardiovascular and renal outcomes in diabetic patients: a systematic review and meta-analysis. Cardiorenal Med.

[10] Neal B, Perkovic V, Mahaffey KW (2017). Canagliflozin and cardiovascular and renal events in type 2 diabetes. N Engl J Med.

[11] Khan MA, Hashim MJ, King JK, Govender RD, Mustafa H, Al Kaabi J (2020). Epidemiology of type 2 diabetes - global burden of disease and forecasted trends. J Epidemiol Glob Health.

[12] Page MJ, McKenzie JE, Bossuyt PM (2021). The PRISMA 2020 statement: an updated guideline for reporting systematic reviews. BMJ.

[13] (2021). RoB 2: A revised Cochrane risk-of-bias tool for randomized trials. https://methods.cochrane.org/bias/resources/rob-2-revised-cochrane-risk-bias-tool-randomized-trials.

[14] Wanner C, Inzucchi SE, Lachin JM (2016). Empagliflozin and progression of kidney disease in type 2 diabetes. N Engl J Med.

[15] Perkovic V, Jardine MJ, Neal B (2019). Canagliflozin and renal outcomes in type 2 diabetes and nephropathy. N Engl J Med.

[16] Phrommintikul A, Wongcharoen W, Kumfu S, Jaiwongkam T, Gunaparn S, Chattipakorn S, Chattipakorn N (2019). Effects of dapagliflozin vs vildagliptin on cardiometabolic parameters in diabetic patients with coronary artery disease: a randomised study. Br J Clin Pharmacol.

[17] Bonora BM, Vigili de Kreutzenberg S, Avogaro A, Fadini GP (2019). Effects of the SGLT2 inhibitor dapagliflozin on cardiac function evaluated by impedance cardiography in patients with type 2 diabetes. Secondary analysis of a randomized placebo-controlled trial. Cardiovasc Diabetol.

[18] Petrie MC, Verma S, Docherty KF (2020). Effect of dapagliflozin on worsening heart failure and cardiovascular death in patients with heart failure with and without diabetes. JAMA.

[19] Fuchigami A, Shigiyama F, Kitazawa T (2020). Efficacy of dapagliflozin versus sitagliptin on cardiometabolic risk factors in Japanese patients with type 2 diabetes: a prospective, randomized study (DIVERSITY-CVR). Cardiovasc Diabetol.

[20] Packer M, Anker SD, Butler J (2020). Cardiovascular and renal outcomes with empagliflozin in heart failure. N Engl J Med.

[21] Cannon CP, Pratley R, Dagogo-Jack S (2020). Cardiovascular outcomes with ertugliflozin in type 2 diabetes. N Engl J Med.

[22] Steinberg J, Carlson L (2021). Type 2 diabetes Therapies: a STEPS approach. Am Fam Physician.

[23] Laleman N, Henrard S, van den Akker M, Goderis G, Buntinx F, Van Pottelbergh G, Vaes B (2018). Time trends in statin use and incidence of recurrent cardiovascular events in secondary prevention between 1999 and 2013: a registry-based study. BMC Cardiovasc Disord.

[24] (2021). National Diabetes Statistics Report: Estimates of Diabetes and Its Burden in the United States. https://www.cdc.gov/diabetes/pdfs/data/statistics/national-diabetes-statistics-report.pdf.

[25] (2021). Heart Disease Facts. https://www.cdc.gov/heartdisease/facts.htm.

[26] Lopaschuk GD, Verma S (2020). Mechanisms of cardiovascular benefits of sodium glucose co-transporter 2 (SGLT2) Inhibitors: a state-of-the-art review. JACC Basic Transl Sci.

[27] Sattar N (2013). Revisiting the links between glycaemia, diabetes and cardiovascular disease. Diabetologia.

[28] Ferrannini E, Baldi S, Frascerra S, Astiarraga B, Barsotti E, Clerico A, Muscelli E (2017). Renal handling of ketones in response to sodium-glucose cotransporter 2 inhibition in patients with type 2 diabetes. Diabetes Care.

[29] Hallow KM, Helmlinger G, Greasley PJ, McMurray JJ, Boulton DW (2018). Why do SGLT2 inhibitors reduce heart failure hospitalization? A differential volume regulation hypothesis. Diabetes Obes Metab.

[30] AbouEzzeddine OF, Kemp BJ, Borlaug BA (2019). Myocardial energetics in heart failure with preserved ejection fraction. Circ Heart Fail.

[31] Bedi KC Jr, Snyder NW, Brandimarto J (2016). Evidence for intramyocardial disruption of lipid metabolism and increased myocardial ketone utilization in advanced human heart failure. Circulation.

[32] Lee TM, Chang NC, Lin SZ (2017). Dapagliflozin, a selective SGLT2 Inhibitor, attenuated cardiac fibrosis by regulating the macrophage polarization via STAT3 signaling in infarcted rat hearts. Free Radic Biol Med.

[33] Byrne NJ, Matsumura N, Maayah ZH (2020). Empagliflozin blunts worsening cardiac dysfunction associated with reduced nlrp3 (nucleotide-binding domain-like receptor protein 3) inflammasome activation in heart failure. Circ Heart Fail.

[34] Lim VG, Bell RM, Arjun S, Kolatsi-Joannou M, Long DA, Yellon DM (2019). SGLT2 inhibitor, canagliflozin, attenuates myocardial infarction in the diabetic and nondiabetic heart. JACC Basic Transl Sci.

[35] Verma S, Garg A, Yan AT (2016). Effect of empagliflozin on left ventricular mass and diastolic function in individuals with diabetes: an important clue to the EMPA-REG OUTCOME trial?. Diabetes Care.

